# Chemerin Overexpression in the Liver Protects against Inflammation in Experimental Non-Alcoholic Steatohepatitis

**DOI:** 10.3390/biomedicines10010132

**Published:** 2022-01-07

**Authors:** Rebekka Pohl, Susanne Feder, Elisabeth M. Haberl, Lisa Rein-Fischboeck, Thomas S. Weiss, Marlen Spirk, Astrid Bruckmann, Nichole McMullen, Christopher J. Sinal, Christa Buechler

**Affiliations:** 1 Department of Internal Medicine I, Regensburg University Hospital, 93053 Regensburg, Germany; becky-pohl@web.de (R.P.); feder.susanne@gmx.de (S.F.); haberl.elisabeth@gmx.de (E.M.H.); lisa.rein-fischboeck@gmx.de (L.R.-F.); marlen.spirk@stud.uni-regensburg.de (M.S.); 2 Children’s University Hospital (KUNO), Regensburg University Hospital, 93053 Regensburg, Germany; thomas.weiss@ukr.de; 3 Biochemistry Center Regensburg (BZR), Laboratory for RNA Biology, University of Regensburg, 93053 Regensburg, Germany; astrid.bruckmann@vkl.uni-regensburg.de; 4 Department of Pharmacology, Dalhousie University, Halifax, NS B3H 4R2, Canada; nichole.mcmullen@Dal.Ca (N.M.); christopher.sinal@Dal.Ca (C.J.S.)

**Keywords:** peripheral blood mononuclear cells, chemerin activity, hepatic inflammation, hepatic stellate cells

## Abstract

Non-alcoholic steatohepatitis (NASH) is marked by macrophage infiltration and inflammation. Chemerin is a chemoattractant protein and is abundant in hepatocytes. The aim of this study was to gain insight into the role of hepatocyte-produced prochemerin in NASH. Therefore, mice were infected with adeno-associated virus 8 to direct hepatic overexpression of prochemerin in a methionine–choline deficient dietary model of NASH. At the end of the study, hepatic and serum chemerin were higher in the chemerin-expressing mice. These animals had less hepatic oxidative stress, F4/80 and CC-chemokine ligand 2 (CCL2) protein, and mRNA levels of inflammatory genes than the respective control animals. In order to identify the underlying mechanisms, prochemerin was expressed in hepatocytes and the hepatic stellate cells, LX-2. Here, chemerin had no effect on cell viability, production of inflammatory, or pro-fibrotic factors. Notably, cultivation of human peripheral blood mononuclear cells (PBMCs) in the supernatant of Huh7 cells overexpressing chemerin reduced CCL2, interleukin-6, and osteopontin levels in cell media. CCL2 was also low in RAW264.7 cells exposed to Hepa1–6 cell produced chemerin. In summary, the current study showed that prochemerin overexpression had little effect on hepatocytes and hepatic stellate cells. Of note, hepatocyte-produced chemerin deactivated PBMCs and protected against inflammation in experimental NASH.

## 1. Introduction

Non-alcoholic fatty liver disease (NAFLD) is a major cause of chronic liver injury with a pathophysiology that is closely linked to obesity and insulin resistance [1,2]. Hepatic steatosis is relatively common in the Western population and may progress to non-alcoholic steatohepatitis (NASH) [1,3]. Innate immune mechanisms have an eminent role in NASH. Liver inflammation drives hepatic fibrosis, and a blockade of macrophage infiltration prevented activation of hepatic stellate cells and suppressed fibrogenesis [4].

The chemoattractant protein, chemerin, induces chemotaxis in macrophages, dendritic cells, and natural killer cells and is highly expressed in hepatocytes [5,6,7,8,9]. Chemerin is released as inactive prochemerin, and C-terminal proteolysis generates biologically active and inactive variants [10].

In mice, prochemerin has 162 amino acids and is referred to as muChem-162 in this manuscript. Human prochemerin, which is one amino acid longer, is designated as huChem-163. MuChem-156 (and the human variant with one more amino acid, huChem-157) is the most well-studied chemerin variant, which exhibits high chemotactic activity mediated by binding and activation of chemokine-like receptor 1 (CMKLR1) [11,12]. G protein-coupled receptor 1 (GPR1) binds chemerin with an affinity comparable to CMKLR1 [13]; however, the biological functions of this receptor are less well characterized.

In addition to a role of chemerin in chemotaxis, chemerin has been implicated in glucose metabolism; however, there is conflicting data in this regard. For example, exogenous chemerin treatment of diabetic db/db mice lowered serum insulin levels and augmented glucose intolerance [14]. In contrast to this model, ubiquitous chemerin knockout reduced glucose-stimulated insulin secretion and induced glucose intolerance while hepatic chemerin overexpression improved both of these measures [15]. Other studies have reported impaired insulin sensitivity after treatment of skeletal muscle cells with exogenous chemerin [16,17]. Despite these conflicting results, analysis of patient cohorts were largely consistent in reporting positive correlations of serum chemerin with markers of obesity, insulin resistance, and blood pressure [18]. Interestingly, while whole body knockdown of chemerin was associated with lower blood pressure in rats, liver-specific depletion of chemerin was without effect [19]. This supports distinct physiological roles for hepatocyte-processed chemerin versus that derived from white adipose tissues.

At present, few studies have investigated the biological actions and anatomical distribution of chemerin isoforms. While the predominant variant reported in human plasma is full-length prochemerin (huChem-163), huChem-157 and huChem-155 were the most abundant isoforms in human subcutaneous and omental adipose tissue, respectively [20]. In murine epididymal fat, muChem-155 (the human homolog is huChem-156) was the dominant variant [21]. Thus, it is likely that there is considerable variability not only in the absolute amount but also the spectrum of isoforms in different tissues. However, the majority of research to date has focused on huChem-157 (muChem-156) and the role of other chemerin variants, and tissue-specific processing of chemerin remains largely unknown. Moreover, the vast majority of studies have relied on immunodetection of total chemerin protein levels, which may not correspond to biological activity. For example, studies of serum chemerin protein levels reported a lack of concordance with ex-vivo measured activation of the receptors CMKLR1 and GPR1 in obesity [22,23]. Likewise, levels of C-terminally processed inactive chemerin isoforms were induced in the plasma of the obese [20].

Activation of CMKLR1 and GPR1 by chemerin can be quantified by the beta-arrestin 2 Tango assay [24]. Chemerin isoforms, which do not induce beta-arrestin 2 recruitment, are so far referred to as inactive variants [10]. It is quite possible that these isoforms induce signaling pathways, which have not been identified so far.

The pathology of NASH is highly complex and macrophage infiltration is a hallmark of liver injury. During NASH development, CC-chemokine ligand (CCL)2 and its receptor, CC-chemokine receptor (CCR2), were upregulated in the liver and contributed to macrophage accumulation and inflammation [25]. Activation of liver-resident macrophages as well as hepatic stellate cells promotes hepatic fibrosis and progression of NASH [25].

Despite the liver being a major anatomical site of chemerin expression, most studies to date have focused on adipose tissue-derived chemerin. Moreover, CMKLR1 and GPR1 as well as the non-signaling chemerin receptor C-C chemokine receptor-like 2 (CCRL2), are expressed in the liver [6,26,27,28,29]. This suggests that the liver is both an abundant source of chemerin and a target for the biological actions of this protein. The diverse functions of chemerin in metabolism as well as its role as an immune cell attractant further indicate a possible pathophysiological function of chemerin in inflammatory liver disorders such as NASH [10].

A recent study described a protective role for huChem-157 injection in a high-fat diet model of NASH [30]. Hepatic steatosis, hepatocyte ballooning, and lobular and portal inflammation were all improved [30]. Notably, recombinant chemerin induced the hepatic mRNA expression of chemerin and CMKLR1 [30]. In the experimental model described above endogenous chemerin protein was low in the NASH liver [30]. This finding has been contradicted by other studies which reported that hepatic chemerin expression was either not changed or induced in the murine NASH liver [31]. Results reported on the hepatic expression of chemerin in NAFLD patients are also inconsistent [32,33,34].

The high-fat diet NASH model is confounded by weight gain, and various adipose tissue produced factors, such as chemerin, increase in serum [35]. The methionine–choline-deficient (MCD) diet-fed mice lose body weight and have serum chemerin levels similar to the respective controls [6].

The aim of this study was to investigate the role of hepatocyte-produced prochemerin in NASH. Consequently, mice overexpressing muChem-162 in the liver were fed an MCD diet. Remarkably, muChem-162 protected against NASH-associated oxidative stress and inflammation.

## 2. Materials and Methods

### 2.1. Quantification of Metabolites

Triglyceride concentrations were measured using the Triglyceride Quantification Kit (BioVision, Milpitas, CA, USA, order number: K622-100-BV). Liver cholesterol was determined by an assay from Diaglobal (Berlin, Germany, order number: CHO 013). Insulin ELISA was from Mercodia (Uppsala, Sweden, order number: 10-1149-01), and the QuantiChrom Glucose Assay Kit was from Biotrend (Köln, Germany, order number: DIGL-100). Lactate dehydrogenase (LDH) in the supernatants was measured with the Cytotoxicity Detection Kit from Roche (Mannheim, Germany, order number: 11644793001).

### 2.2. Animal Studies

Male C57BL/6J mice were ordered from Charles River (Sulzfeld, Germany) and housed in a 21 ± 1 °C controlled room under a 12 h light–dark cycle. Animals had free access to food and water and were housed with 4–7 mice per cage. Adeno-associated virus 8 (AAV8; 10^11^ per animal) expressing prochemerin (muChem-162, *n* = 7) or empty vector (*n* = 7) were delivered to nine-week-old male mice fed a standard chow via intraperitoneal injection. AAV8 was constructed by SIRION Biotech (Planegg-Martinsried, Germany) as described in [36]. The mouse alpha-fetoprotein enhancer and the mouse minimal albumin promoter controlled the expression of prochemerin. Immediately after injection of AAV8, the mice were kept on an MCD diet (E15653-94) or the respective control diet (E15654-04, Ssniff, Soest, Germany) for two weeks. At the end of the study, mice were killed by CO_2_ asphyxiation followed by cervical dislocation. Blood was collected by cardiac puncture after fasting overnight. Tissues were removed and rapidly frozen in liquid nitrogen and maintained at −80 °C.

### 2.3. Immunoblotting and Histology

Immunoblotting was performed as described [37]. The antibodies used are listed in the Supplementary Materials Table S1. Hematoxylin–Eosin and Sirius Red staining were performed as reported in [37].

### 2.4. Monitoring of Gene Expression by Real-Time RT-PCR

Real-time RT-PCR was conducted as described in [26], and the primers used are listed in Supplementary Materials Table S2.

### 2.5. Peripheral Blood Mononuclear Cells and Cell Lines

Peripheral blood mononuclear cells (PBMCs) from four different donors were ordered from Hepacult GmbH (Regensburg, Germany). These cells were cultivated in the cell media of hepatocytes, which was centrifuged for 5 min at 4000 rpm before being used. The LX-2 human hepatic stellate cell line and hepatocyte cell lines were cultivated as described [38,39]. Cell number and viability were determined using the Countess II FL from Life Technologies (Thermo Fisher Scientific, Waltham, MA, USA). RAW264.7 cells were purchased from the American Type Culture Collection (Manassas, VA, USA) and were cultivated in RPMI medium supplemented with 10% FBS and 1% penicillin/streptomycin.

### 2.6. Tango Assay

Ex-vivo activation of CMKLR1 and GPR1 by serum chemerin was analyzed by the Tango assay as described in [22].

### 2.7. Recombinant Expression of Chemerin Isoforms in Hepatocytes

Polymerase chain reaction to amplify human chemerin cDNA was performed with the forward primer 5’-CGA AAG CTT ATG CGA CGG CTG CTG ATC C-3’ and the reverse primer 5’-CGA CCG CGG TTA GCT GCG GGG CAG G-3’. The resultant cDNA was cloned into the plasmid pcDNA3.1 (Thermo Fisher Scientific, Waltham, MA, USA, order number: V79020). The cutting sites for the restriction enzymes (i.e., HindIII and SacII) are underlined. The DNA sequences of the cloned fragments were confirmed by sequence analysis (GeneArt, Regensburg, Germany). Murine chemerin was produced in Hepa1–6 cells as described in [36].

### 2.8. ELISAs and Cytokine Array

ELISAs to measure IL-6 (order number human: DY206; murine DY406), TNF (order number murine: DY410), CXCL1 (order number murine: DY453), osteopontin (order number human: DY1433), chemerin (order number human: DY2324; order number mouse: DY2325), CCL20 (order number human: DY360) and CCL2 (order number human: DY279; murine: DY479) were from R&D Systems (Wiesbaden, Nordenstadt, Germany), and the alanine aminotransferase (ALT) ELISA was from BIOZOL (Eching, Germany, order number: MBS264717). Proteome Profiler^TM^ Human XL cytokine array (order number: ARY022B) was from R&D Systems and was hybridized with the cell culture medium as recommended by the supplier.

### 2.9. Mass Spectrometry of Chemerin Protein

The mass spectrometry method used to identify chemerin isoforms in the liver was recently described [36].

### 2.10. Immunohistochemistry

Immunohistochemistry was performed by the use of the VECTASTAIN ABC KIT (Vector Laboratories, BIOZOL Diagnostica, Eching, Germany, order number: VEC-PK-4000) and the ImmPACT DAB peroxidase substrate (Vector Laboratories, order number: VEC-SK-4105) The F4/80 antibody used is listed in the Supplementary Materials Table S1 and was 1:250-fold diluted.

### 2.11. Statistical Analysis

When not indicated otherwise in the figure legend, data are given as box plots. The statistical tests used were the Mann–Whitney U test (SPSS Statistics 25.0 program) or the Student’s *t*-test (MS Excel). A value of *p* < 0.05 was regarded as significant.

## 3. Results

### 3.1. MuChem-162 Expression in the Liver Increased Hepatic and Systemic Chemerin

**Figure 1:**
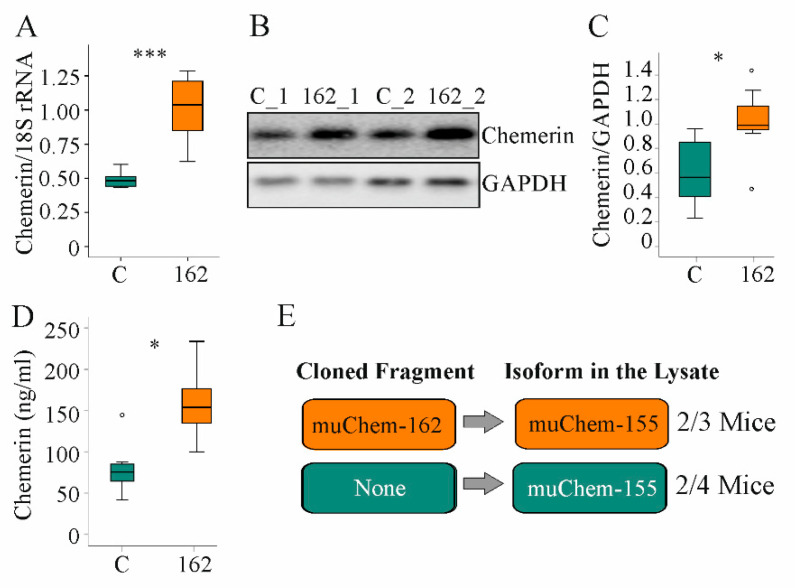
Overexpression of muChem-162 in the liver increased chemerin in mice fed an methionine–choline deficient (MCD) diet for 2 weeks: (**A**) *Chemerin* mRNA in the liver of mice infected with the control AAV8 (**C**) and mice infected with muChem-162-producing AAV8 (*n* = 7 mice per group); (**B**) Immunoblot of hepatic chemerin protein of two control-infected mice (i.e., C_1 and C_2) and two muChem-162-expressing mice (i.e., 162_1 and 162_2); (**C**) Quantification of hepatic chemerin protein (*n* = 7 mice per group); (**D**) Serum chemerin (*n* = 7 mice per group); (**E**) Chemerin isoforms in the liver of control-infected mice and muChem-162-expressing mice. Mann–Whitney U test, * *p* < 0.05, *** *p* < 0.001. Outliers *=* values between 1.5 and 3 times the interquartile range are shown as circles.

Here, the role of hepatocyte-expressed prochemerin in NAFLD was studied. Therefore, immediately after AAV8 injection, mice were fed the MCD diet for two weeks. Hepatic chemerin mRNA and protein expression increased in AAV8-muChem-162-infected mice (Figure 1A–C). Serum chemerin was significantly elevated (Figure 1D).

Chemerin is proteolyzed at the C-terminal end to produce several unique isoforms [10]. To clarify which chemerin isoforms exist in the liver, mass spectrometric analysis was conducted. Thereby, the active isoform muChem-155 was identified in the liver of two out of three mice with muChem-162 overexpression and in the liver of two out of four mice transfected with the control AAV8 virus (Figure 1E). The protein used for mass spectrometry was digested with trypsin, which cleaved murine chemerin after amino acid 136, 157, and 160. This approach is appropriate for detecting muChem-156 and smaller isoforms but not muChem-157 or prochemerin. This preliminary analysis showed that biologically active isoforms are detectable in the murine NASH liver.

### 3.2. MuChem-162 Expression in the Liver Did Not Affect Glucose and Lipid Levels

**Figure 2:**
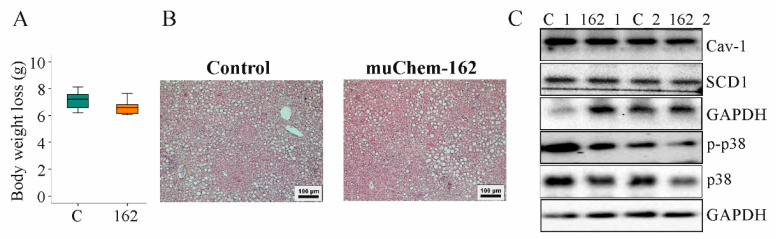
Overexpression of muChem-162 in the liver had no effect on body weight loss, hepatic steatosis, and chemerin signaling in mice fed an methionine–choline deficient (MCD) diet for 2 weeks: (**A**) Body weight loss (*n* = 7 mice per group; Mann–Whitney U test); (**B**) Hematoxylin-and-eosin-stained liver; (**C**) Immunoblot analysis of caveolin-1 (Cav-1), stearoyl-CoA-desaturase (SCD) 1, p38 protein, and its phosphorylated form (*p*-) in the liver of two control-infected mice (i.e., C_1 and C_2) and two muChem-162-expressing mice (i.e., 162_1 and 162_2).

Body weight and body weight loss during the study period were not changed by muChem-162 (Figure 2A and Supplementary Materials Figure S1). Liver weight and spleen weight were comparable in both groups (Table 1).

Serum adiponectin, glucose, and insulin were similar between the mice groups (Table 1). Hepatic cholesterol and triglycerides were unchanged by muChem-162 overexpression (Table 1). Histological images show a similar severity of liver steatosis in both mouse groups (Figure 2B). Caveolin-1 and stearoyl-CoA-desaturase (SCD)1 protein, which are downregulated in NAFLD liver [40,41], were comparable between both groups (Figure 2C and Supplementary Materials Table S3).

Phosphorylation of p38 mitogen-activated protein kinase, a downstream target of chemerin [42,43], was similar in the livers of all mice (Figure 2C and Supplementary Materials Table S3).

### 3.3. MuChem-162 Expression in the Liver Improved Hepatic Inflammation

**Figure 3:**
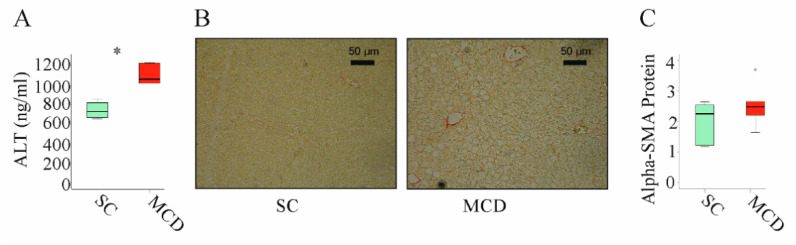
Two weeks of an MCD diet induced serum alanine aminotransferase (ALT) and liver steatosis but not liver fibrosis: (**A**) ALT measured in the serum of mice fed standard chow (SC) or an methionine–choline deficient (MCD) diet for 2 weeks; (**B**) Sirius-red-stained liver of mice fed an SC or an MCD diet for 2 weeks; (**C**) Alpha-smooth muscle actin (alpha-SMA) protein in the liver of mice fed an SC or an MCD diet for 2 weeks (*n* = 4–5 mice per group). Mann–Whitney U test, * *p* < 0.05. Outliers *=* values between 1.5 and 3 times the interquartile range are shown as circles.

NASH is associated with higher expression of inflammatory and fibrotic genes [44]. It is important to note that the feeding of an MCD diet for 2 weeks increased alanine aminotransferase (ALT) in serum (Figure 3A). Moreover, these animals develop liver steatosis (Figure 3B). These mice did not develop liver fibrosis as was analyzed by Sirius red staining (Figure 3B) [41] and expression of alpha-smooth muscle actin (SMA) protein, which was not upregulated (Figure 3C).

**Figure 4:**
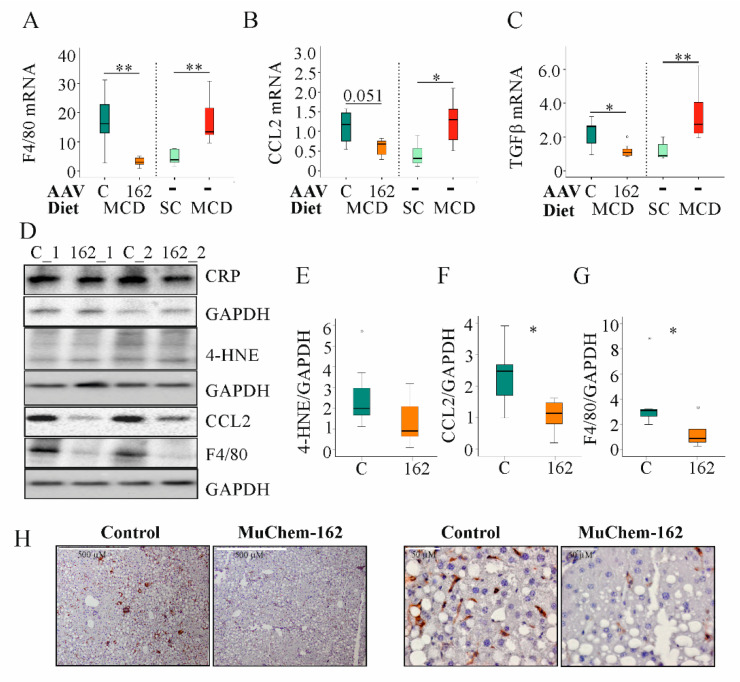
Overexpression of muChem-162 normalized *F4/80*, *CC-chemokine ligand* (CCL)2, and *transforming growth factor* (TGF)*β* mRNA levels in the liver of methionine–choline deficient (MCD)-diet-fed mice and reduced 4-hydroxynonenal (4-HNE), CCL2, and F4/80 protein levels: (**A**) *F4/80* mRNA in the liver of mice infected with the control AAV8 or muChem-162-producing AAV8 fed an MCD diet. Data for non-infected mice fed standard chow (SC) or an MCD diet are shown in parallel on the right side of the dotted line. (**B**) CCL2 mRNA in the liver of these mice. The number in the figure is the *p*-value. (**C**) *TGFβ* mRNA in the liver of these mice. (**D**) CRP, 4-HNE, CCL2, and F4/80 protein in the liver of two control-infected mice (i.e., C_1 and C_2) and two muChem-162-expressing mice (i.e., 162_1 and 162_2) fed an MCD diet. (**E**) Quantification of the 4-HNE. (**F**) Quantification of the CCL2 protein. (**G**) Quantification of the F4/80 protein. Protein levels were normalized to glyceraldehyde 3 phosphate dehydrogenase (GAPDH) (*n* = 6–7 mice per group). Mann–Whitney U test. (**H**) Immunohistochemistry of F4/80 in the liver. * *p* < 0.05, ** *p* < 0.01. Outliers *=* values between 1.5 and 3 times the interquartile range are shown as circles, and values more than 3 times the interquartile range are shown as stars.

In the MCD model used here, *F4/80*, *CCL2*, and *transforming growth factor* (TGF) β mRNA increased after 2 weeks of feeding (Figure 4A–C).

Overexpression of muChem-162 in the liver was associated with reduced hepatic mRNA levels of *CCL2*, *F4/80*, *CD38*, *CD68*, and *tumor necrosis factor* (TNF), all of which are expressed primarily by immune cells. Expression of *CD163*, which is a specific marker of M2 macrophages [45], increased (Table 2). Natural killer cell-expressed *Ncr1* was reduced, whereas *Ly49C* did not change (Table 2). The genes for the profibrotic proteins *TGFβ*, *connective tissue growth factor* (CTGF), *alpha-SMA*, and *Col1a1* were all diminished with hepatic muChem-162 overexpression (Table 2). *IL-6* mRNA expression also declined but this effect was not significant (Table 2).

To determine if muChem-162 overexpression can normalize NASH-associated upregulation of *F4/80*, *CCL2*, and *TGFβ* mRNA, gene expression of age-matched male mice fed either the control chow or the MCD diet for two weeks was compared with the mRNA levels of the AAV8-infected mice (Table 2). Comparison of both data sets revealed that muChem-162 in fact normalized the mRNA levels of these genes (Figure 4A–C).

The lipid peroxidation product, 4-hydroxynonenal, was higher in the liver of control-infected mice, indicating reduced oxidative stress in mice with muChem-162 overexpression (Figure 4D,E). Hepatic inflammation is another characteristic of NASH. Whereas hepatic C-reactive protein (CRP) levels did not differ between the control and muChem-162-infected mice, CCL2 protein was reduced in the latter group (Figure 4D,F). Accordingly, the macrophage specific protein, F4/80, was less expressed in the liver of mice overexpressing chemerin (Figure 4D,G,H).

### 3.4. Overexpression of MuChem-162 in Hepatocytes Did Not Change Expression of Inflammatory and Fibrotic Genes

**Figure 5:**
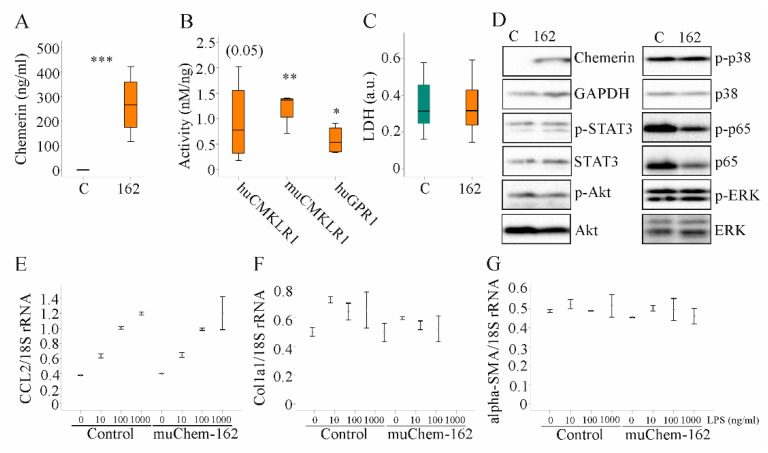
Overexpression of muChem-162 had no effect on inflammatory and profibrotic molecules in Hepa1–6 cells: (**A**) Chemerin in the supernatant of Hepa1–6 cells transfected with the insert-less (C) or muChem-162-coding plasmids (162) (*n* = 4); (**B**) Activation of human and murine chemokine-like receptor 1 (CMKLR1) and human G protein-coupled receptor 1 (GPR1) by media of the recombinant cells relative to chemerin protein levels. Control-transfected cell media were not active (*n* = 4); (**C**) Lactate dehydrogenase (LDH) in the cell media (*n* = 13); (**D**) Immunoblot of chemerin, signal transducer and activator of transcription 3 (STAT3), Akt, p38 kinase, p65, and extracellular-signal regulated kinase (ERK) and their phosphorylated (p-) forms; (**E**) *CCL2* mRNA in Hepa1–6 cells transfected with the control vector or the plasmid to express muChem-162, and cells were incubated with increasing concentrations of LPS (*n* = 2); (**F**) *Collagen* (Col)1a1 mRNA in these cells (*n* = 2); (**G**) *Alpha-smooth muscle actin* (alpha-SMA) mRNA in these cells (*n* = 2). In (**E**–**G**), the mean values +/− standard deviations are shown. Student’s t-test, * *p* < 0.05, ** *p* < 0.01, *** *p* < 0.001.

In vitro studies were carried out to characterize the pathways that contribute to the anti-inflammatory effect of hepatocyte-produced chemerin in experimental NASH. Overexpression of muChem-162 in the murine hepatocyte cell line Hepa1–6 cells, which do not express detectable chemerin, increased cellular chemerin (protein as determined by immunoblot analysis was (264 (109–272) in control-transfected cells and 5572 (4649–5718) in muChem-162-expressing Hepa1–6 cells; *p* = 0.002) and soluble chemerin protein levels (Figure 5A). Mass spectrometry detected muChem-156 and muChem-155 in the cell media of muChem-162 overexpressing cells (data not shown), which are both active isoforms [10]. Recombinant chemerin in the supernatant activated murine and human CMKLR1, and human GPR1 (Figure 5B). This shows that hepatocytes processed muChem-162 and produced active chemerin isoforms.

Median muCMKLR1 activity was 1.36 nM/ng recombinant chemerin when muChem-162 was overexpressed in Hepa1–6 cells and was 2.56 nM/ng recombinant chemerin when muChem-156 was overexpressed (Figure 5B and Supplementary Materials Table S4). This suggests that only approximately 50% of the recombinant muChem-162 was processed by the Hepa1–6 cells. Regarding huGPR1 mediated beta-arrestin 2 recruitment, muChem-162 overexpression produced an approximately three-fold lower activity per ng recombinant chemerin when compared to cells’ overexpressing muChem-156 (Supplementary Materials Table S4). An approximately five-fold difference was noticed for activation of huCMKLR1, where the median activity of muChem-156 was nearly 4.0 nM/ng and muChem-162 was 0.77 nM/ng recombinant chemerin (Figure 5B and Supplementary Materials Table S4).

Recombinant muChem-162 overexpression was not cytotoxic (Figure 5C) and did not affect cell proliferation (data not shown). The overexpressed protein did not change phosphorylation of the chemerin signaling molecules’ signal transducer and activator of transcription 3 (STAT3), Akt, p38 kinase, p65, and extracellular-signal regulated kinase (ERK) (Figure 5D and Supplementary Materials Table S5). MuChem-162 overexpression did not regulate mRNA levels of *CCL2*, *collagen* (Col)1a1, or *alpha-SMA* in the hepatocytes (Figure 5E–G). Lipopolysaccharide (LPS) dose-dependently induced *CCL2* mRNA, and this induction was not modified by muChem-162 overexpression (Figure 5E). CCL2, TNF, and the chemokine CXCL1 were also measured in Hepa1–6 cell media. Overexpression of muChem162 had no effect on these protein levels (Supplementary Materials Table S6). Thus, muChem-162 overexpression had no effect on cell viability, central signaling pathways, the chemokines CCL2 and CXCL1, the cytokine TNF, and the expression of the genes described above.

### 3.5. Overexpression of HuChem-163 in LX-2 Cells Did Not Change Proliferation and Expression of Profibrotic Markers

**Figure 6:**
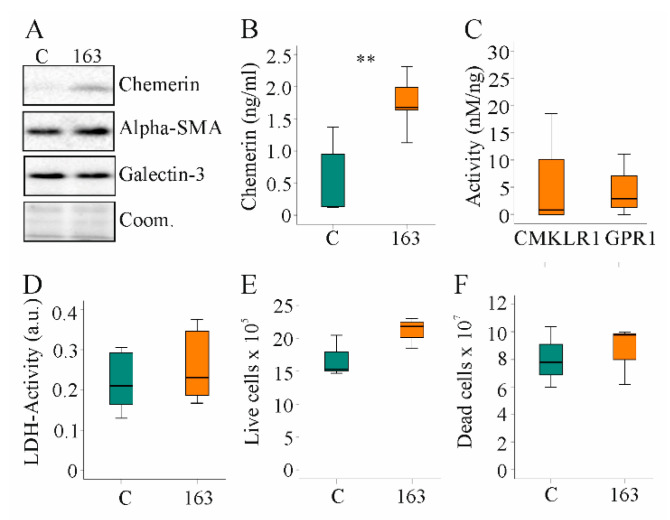
Overexpression of huChem-163 in human LX-2 cells: (**A**) Immunoblot of chemerin, alpha-smooth muscle actin (alpha-SMA), and galectin-3 in LX-2 cells overexpressing huChem-163; (**B**) Chemerin in the supernatant of these cells (*n* = 4); (**C**) Activation of human chemokine-like receptor 1 (CMKLR1) and human G protein-coupled receptor 1 (GPR1) by the media of the recombinant cells relative to total cell media chemerin. Control-transfected cell supernatant was not active, and overexpression of huChem-163 did not significantly increase CMKLR1 or GPR1 activity (*n* = 4); (**D**) Lactate dehydrogenase (LDH) activity (*n* = 4; arbitrary units: a.u.); (**E**) Number of live cells (*n* = 3); (**F**) Number of dead cells (*n* = 3). Student’s *t*-test, ** *p* < 0.01.

Hepatic stellate cell activation and proliferation is a central process in chronic liver diseases [46]. The human hepatic stellate cell line, LX-2, expressed low endogenous levels of chemerin. Both cellular and soluble chemerin were induced in LX-2 cells by transfection with a plasmid to express huChem-163 (Figure 6A,B). Activation of huCMKLR1 and huGPR1 was not significantly increased by media of cells producing recombinant huChem-163 (Figure 6C). Median huCMKLR1 activity was 14.7 nM/ng recombinant chemerin when huChem-157 was overexpressed [39] and was 0.8 nM/ng when huChem-163 was overexpressed showing an approximately 17-fold lower activity of recombinant huChem-163. Regarding huGPR1-mediated beta-arrestin 2 recruitment, huChem-163 overexpression led to an approximately two-fold lower activity compared to huChem-157 (median activity was 5.6 nM/ng) (Figure 6C).

Activated hepatic stellate cells acquire the ability to proliferate rapidly and produce profibrotic proteins [46]. Expression of alpha-SMA and galectin-3 proteins was unchanged by endogenous huChem-163 expression (Figure 6A). HuChem-163 overexpression in the hepatic stellate cell line LX-2 did not affect the LDH activity or number of live and dead cells (Figure 6D–F). Overexpression of huChem-163 in the LX-2 cell line did not significantly change CCL2, osteopontin, or IL-6 levels in cell media (Supplementary Materials Table S7).

### 3.6. Media of Hepatocytes Overexpressing HuChem-163 Lowered CCL2, IL-6, and Osteopontin in Supernatants of PBMCs and CCL2 in RAW264.7 Cell Media

**Figure 7:**
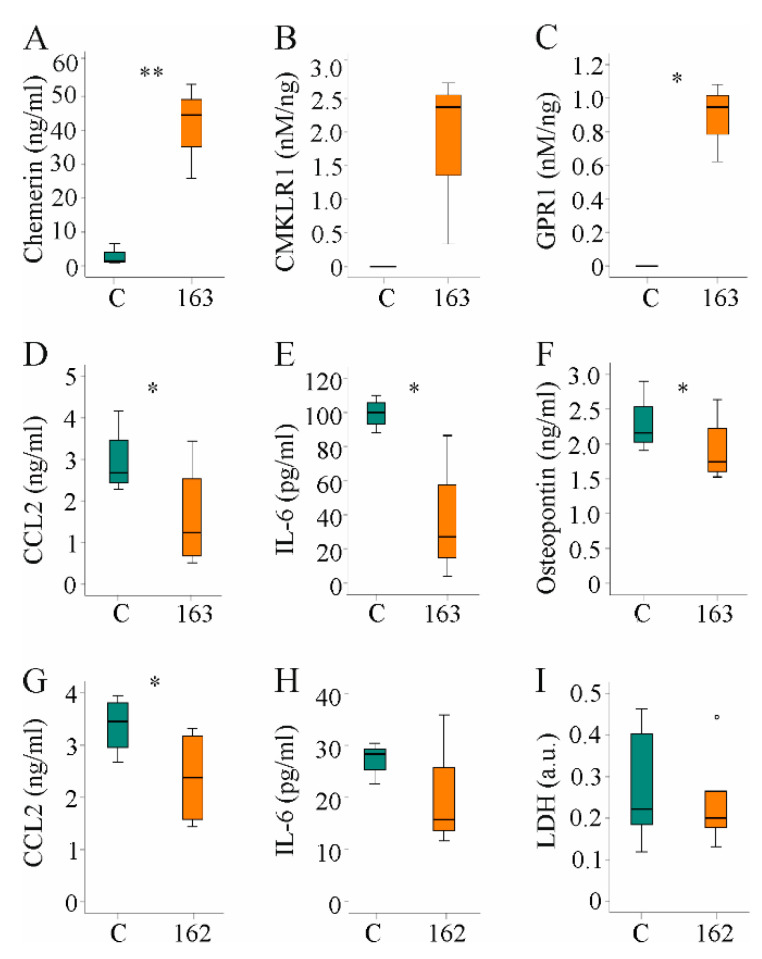
Hepatocyte supernatant of huChem-163 expressing Huh7 cells lowered inflammatory proteins in human peripheral blood mononuclear cells (PBMC) media and supernatant of Hepa1–6 cells reduced CC-chemokine ligand 2 (CCL2) in RAW264.7 cell media: (**A**) Chemerin in the supernatant of Huh7 cells transfected with an insert-less plasmid (**C**) or a plasmid to express huChem-163 (*n* = 4); (**B**) Chemokine-like receptor 1 (CMKLR1) activation by the supernatant of Huh7 cells transfected with an insert-less plasmid (**C**) or a plasmid to express huChem-163 relative to total cell media chemerin (*n* = 3); (**C**) G protein-coupled receptor 1 (GPR1) activation in these cells (*n* = 3); (**D**) Cell media CCL2 levels of PBMCs cultivated in the supernatant of Huh7 cells transfected with a control plasmid or overexpressing huChem-163 (*n* = 4); (**E**) Interleukin (IL)-6 and (**F**) osteopontin in the media of these PBMCs (*n* = 4); (**G**) Cell media CCL2 levels of RAW264.7 cells cultivated in the supernatant of Hepa1–6 cells overexpressing muChem-162 (*n* = 4); (**H**) IL-6 in these cell media (*n* = 3); (**I**) Lactate dehydrogenase (LDH) in these cell media (*n* = 6). Student’s *t*-test, * *p* < 0.05, ** *p* < 0.01. Outliers *=* values between 1.5 and 3 times the interquartile range are shown as circles.

The results described above indicate that the protective effect of muChem-162 in the context of NASH was most likely not mediated by affecting hepatocyte and/or hepatic stellate cell function. Immune cells are pivotal mediators of liver inflammation and fibrosis [47], and the effect of hepatocyte-released chemerin on human PBMCs was analyzed. Huh7 cells were transfected with a plasmid to express huChem-163 and soluble chemerin was induced (Figure 7A). Activation of CMKLR1 and GPR1 was enhanced by supernatant of huChem-163-producing cells, and this was significant for GPR1 (Figure 7B,C). Mass spectrometry identified huChem-156 in cell media of huChem163 overexpressing Huh7 cells but not the control cells (data not shown).

Median CMKLR1 activity was 3.7 nM/ng recombinant chemerin when huChem-157 was overexpressed [38] and was 2.4 nM/ng when huChem-163 was overexpressed (Figure 7B), showing an approximately 25%-fold lower activity upon expression of recombinant huChem-163. The GPR1 median activity was 2.0 nM/ng in huChem-157 overexpressing cells [38], and 0.94 nM/ng in huChem-163 overexpressing cells (Figure 7C).

Cultivation of human PBMCs in the presence of supernatant from control-infected or huChem-163-expressing Huh7 cells for 24 h showed that huChem-163 lowered CCL2, IL-6, and osteopontin levels in the cell media (Figure 7D–F). Here, it is important to note that CCL2 and IL-6 were not detectable in supernatants of Huh7 cells (data not shown). The mean osteopontin concentration was 1.9 ng/mL in the cell media of control-transfected Huh7 cells and did not change in huChem-163 overexpressing Huh7 cells (Supplementary Materials Table S8).

PBMCs are a mixture of different immune cells such as macrophages and T cells. To evaluate an effect of chemerin on macrophages, RAW264.7 cells were cultivated for 24 h in cell media of prochemerin overexpressing Hepa1–6 cells. CCL2 declined in cell media and a similar trend was observed for IL-6 (Figure 7G,H). To exclude that lower levels of these molecules were related to toxic effects, LDH was also analyzed. Similar levels in cell media of RAW264.7 cultivated in supernatant of control-transfected or prochemerin-producing Hepa1–6 cells excluded an effect on cell viability (Figure 7I).

**Figure 8:**
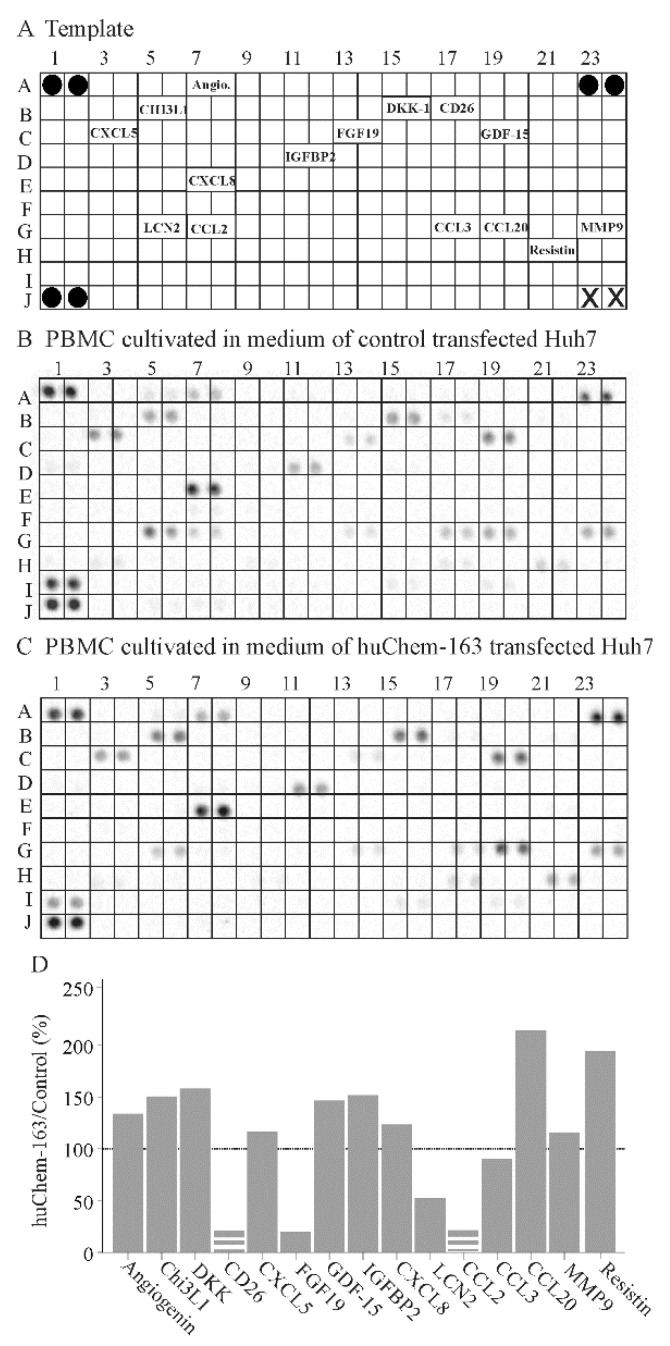
Cytokine array of peripheral blood mononuclear cells (PBMC)s exposed to supernatant of huChem-163 expressing or control-transfected Huh7 cells: (**A**) Template of the array. The soluble factors detected are given at the respective position of the array. Black circles represent the reference spots and X the positions where no antibody was spotted. (**B**) Array hybridized with media of PBMCs cultivated in supernatant of control-transfected Huh7 cells. (**C**) Array hybridized with media of PBMCs cultivated in supernatant of huChem-163 expressing Huh7 cells. (**D**) Arrays were quantified with ImageJ, and the ratios of huChem-163/control stimulated cells are shown. The broken bars were used when the respective cytokine was not detectable on the array shown in (**C**) and the extent of downregulation could not be calculated. The dotted line marks 100%. *n* = 1. Angiogenin = Angio.

To further address the effect of hepatocyte-produced prochemerin on immune cells, a cytokine array was hybridized with the cell media of PBMCs cultivated in supernatant from control-infected or huChem-163 expressing Huh7 cells. This preliminary investigation showed that PBMCs exposed to media of huChem-163 expressing Huh7 cells had lower levels of CD26, fibroblast growth factor 19 (FGF19), lipocalin 2, and CCL2, whereas CCL20 and resistin were induced (Figure 8B–D). An upregulation of CCL20 could not be confirmed by ELISA (Supplementary Materials Figure S2). Moreover, T-cell specific cytokines, such as interferon gamma (position on the array: D9, D10), IL-4 (position on the array: E1, E2), IL-17 (position on the array: E21, 22), and granulocyte macrophage colony stimulating factor (position on the array: C21, C22) [48], were not detected by the array (Figure 8A–C).

## 4. Discussion

The present study demonstrated that hepatic overexpression of prochemerin (muChem-162) protected against the hepatic accumulation of macrophages and liver inflammation in a preclinical NASH model. Consistent with this, prochemerin produced by hepatocytes lowered levels of CCL2, IL-6, and osteopontin in PBMC culture medium and CCL2 in RAW264.7 cell media. Chemerin overexpression had no role in hepatocyte and hepatic stellate cell function. These findings suggest that chemerin isoforms produced by hepatocytes from prochemerin or prochemerin itself act on immune cells and exert anti-inflammatory activities in the liver.

A recent study showed that intraperitoneal injection of huChem-157 was protective in a high-fat diet model of NASH [30]. Hepatic and serum chemerin protein were approximately two-fold higher in these mice. Application of recombinant huChem-157, moreover, induced chemerin mRNA expression in the liver. It is not clear whether endogenously produced chemerin isoforms, the recombinant chemerin, or both were protective in this model [30]. Animals treated with huChem-157 had reduced food uptake and body weight, and the improved metabolic and liver functions may partly result from reduced body weight gain [30]. Body weight was similarly reduced in both groups of mice studied herein, showing that the protective effects of muChem-162 overexpression in the liver were independent of body weight changes.

Very little is known about the function of chemerin in the liver. Hepatic overexpression of prochemerin in low-density lipoprotein-receptor knockout mice impaired the insulin response of skeletal muscle. However, hepatic insulin sensitivity as well as serum lipids were quite normal [16]. In line with this study, Xie et al. reported dysfunctional mitochondria in skeletal muscle of mice with adenoviral overexpression of murine prochemerin [49]. Contrary to these findings, transgenic mice with hepatic overexpression of human prochemerin exhibited better glucose tolerance and glucose-dependent insulin secretion [15]. The reason for the contradictory roles of chemerin in glucose metabolism is not entirely clear. Chemerin isoform distribution, dosage, duration of the treatment, and metabolic state of the animal model may contribute to these discrepancies [10,50].

The mice studied herein had similar fasting serum glucose and insulin levels when overexpressing muChem-162 as the control-infected animals, showing that glucose metabolism was quite normal. Mice fed the MCD diet do lose body weight [51], and this model is not suited to studying the effect of muChem-162 in glucose homeostasis.

Of note, prochemerin expression in murine and human hepatocytes produced bioactive chemerin isoforms that activated the beta-arrestin 2 pathway. In culture media of Huh7 cells overexpressing prochemerin, mass spectrometry analysis detected huChem-156, and the murine homolog muChem-155 was present in cell media of Hepa1–6 cells. This shows that hepatocytes process prochemerin and produce this active isoform. In the murine liver, muChem-155 was identified. These preliminary data confirm the existence of muChem-155 in the liver but cannot give any information about the quantity or distribution of chemerin isoforms in the liver.

Notably, activation of CMKLR1 and GPR1 by muChem-162/huChem-163 overexpressing cells was lower in comparison to muChem-156/huChem-157 overexpressing hepatocytes. This shows that recombinant prochemerin was only partly processed by these cells. Whether the cells are just unable to process all of the recombinant protein or whether C-terminal proteolysis of chemerin is tightly regulated has to be evaluated in future studies.

It must be emphasized that liver resident cells, such as Kupffer cells and endothelial cells, may secrete proteases, such as cathepsin L, which cleave prochemerin [10,52]. Cathepsin L contributes to prochemerin activation and is also involved in the production of very short, inactive chemerin variants [10]. Thus, chemerin isoform distribution in prochemerin overexpressing liver has to be quantified by suitable immune assays, which are not commercially available so far.

In addition to hepatocytes, Kupffer cells, hepatic stellate cells, and endothelial cells express CMKLR1 [28,52]. CCRL2 is expressed by endothelial cells and hepatic stellate cells, but it is most likely not produced by hepatocytes [29,53,54]. GPR1 is the least well-studied chemerin receptor. GPR1 mRNA was found in hepatic stellate cells but was not expressed or very low in abundance in hepatocytes and macrophages [29].

Prochemerin released from hepatocytes may be processed by enzymes released from different liver resident cells. Active chemerin variants may exert effects on the different liver cells expressing the chemerin receptors. Interestingly, the short isoform, huChem-125, does not induce chemotaxis but exerts antimicrobial activity [55], and further research has to resolve the biologic roles of the so-called inactive chemerin variants.

In the mice, muChem-162 overexpression had no effect on the already described activation of p38 by chemerin. Activation of p38 was demonstrated for huChem-157 corresponding to muChem-156 [10,30,43]. Low abundance of active chemerin isoforms may be one explanation for this observation. A further possibility is that chronically high levels of active chemerin desensibilizes the respective signaling molecules. Indeed, overexpression of huChem-157 did not increase phosphorylation of p38 and ERK in Huh7 and HepG2 cells [38]. Consistently, recombinant huChem-157 activated Akt in short-term treatment whereas long-term exposure of 4 or 8 h had no effect [42].

Bioactive chemerin was not/little produced in human hepatic stellate LX-2 cells overexpressing huChem-163. Levels of inflammatory and profibrotic proteins were not changed with the overexpression of full-length chemerin. Moreover, proliferation and cell viability of hepatic stellate cells was unaffected by prochemerin overexpression. It is important to note that chemerin concentrations in the supernatants of LX-2 cells were about 100-fold lower than in Hepa1–6 cells. Thus, in the liver, these cells, which seem to express CMKLR1 and GPR1 [28,29], may respond to chemerin isoforms produced by hepatocytes. Future analysis has to investigate the effect of higher chemerin levels on hepatic stellate cell viability and function.

Importantly, hepatocytes producing huChem-163 exerted anti-inflammatory effects in PBMCs. Overexpression of huChem-156 or huChem-157 was ineffective in this regard [38]. This latter finding is in accordance with previous studies reporting that muChem-156/huChem-157 did not improve the inflammatory response of macrophages [38,56,57]. Of note, huChem-155, which does not stimulate beta-arrestin 2 recruitment to CMKLR1 or GPR1, deactivated PBMCs when overexpressed in Huh7 cells [10,38]. This anti-inflammatory effect of chemerin may involve activation of distinct signaling pathways, which still have to be identified.

Interestingly, CCL2, IL-6, and osteopontin were reduced in PBMC media when these cells were cultivated in media derived from huChem-163 overexpressing Huh7 cells. CCL2 and IL-6 were not detected in the supernatants of the Huh7 cells showing that their production by PBMCs is reduced. PBMCs consist of T lymphocytes, natural killer cells, B cells, and monocytes. IL-6, for example, is produced by almost all immune cells, and its expression is induced by other cytokines such as TNF [58]. Thus, immune cell types have to be analyzed separately to clarify the role of chemerin isoforms in PBMC deactivation. To partly address this, RAW264.7 macrophages were cultivated in Hepa1–6 media. CCL2 and IL-6 were lower when Hepa1–6 cells overexpressed muChem-162.

Moreover, a cytokine array was hybridized with PBMC cell media. Levels of T-cell-specific cytokines [48] could, however, not be detected by this approach. CCL2, CD26, and FGF19 were low in abundance in the PBMCs exposed to Huh7 cell-produced prochemerin. FGF19 is expressed by hepatocyte cell lines and CD26 by T cells [52,59]. Whether chemerin has an effect on hepatocyte-produced FGF19 or T-cell expressed CD26 awaits further research. Upregulation of CCL20, as was detected by the cytokine array, was a false positive result and could not be confirmed by ELISA. Most of the cytokines analyzed did not differ between control and huChem-163 exposed PBMCs excluding an unspecific repressive effect on these cells.

The lower synthesis of CCL2 in the murine liver inhibits immune cell infiltration and fibrogenesis [4]. Osteopontin contributes to liver inflammation and fibrosis, and lower hepatic levels protect from NAFLD [60]. IL-6 has various functions and was shown to contribute to hepatic insulin resistance [61]. In the liver of the mice overexpressing muChem-162 CCL2 mRNA and protein were lower than in the liver of controls suggesting that production of this chemokine by immune cells was reduced. Accordingly, F4/80 protein, which is considered a relative specific marker of tissue macrophages [62], was low in the liver of mice with muChem-162 overexpression. Immunohistochemistry, moreover, showed a lower number of F4/80 expressing cells. 

CD163 is a bona fide marker of M2 macrophages [45], and *CD163* mRNA was induced in the liver of muChem-162-expressing mice, indicating a possible switch from M1 to M2 polarized cells. Macrophage polarization involves various signaling pathways [45], and much more work is needed to clarify the role of chemerin herein.

A further unresolved issue is the role of natural killer cells in NASH [63]. Natural killer cells accumulate in the liver of mice fed an MCD diet [63], and a reduced expression of the natural killer cell receptor, *NCR1*, in the liver of mice overexpressing muChem-162 might indicate depletion of natural killer cells from the liver. Natural killer cells produce various cytokines [64], and lower expression of, for example, *TGFβ* in the liver of muChem-162-overexpressing mice may also be caused by a reduced number of hepatic immune cells.

This study has limitations. The MCD model studied did not develop fibrosis and further experiments are required to resolve this issue. In consideration that Huh7 released chemerin has anti-inflammatory effects, it is well possible that muChem-162 overexpression has effects in the healthy liver which was not studied herein. Moreover, the bioactivity of the recombinant chemerin was analyzed with regard to beta-arrestin 2 recruitment, though further chemerin-responsive pathways have been described [10,43]. The bioactivity of chemerin in the mouse liver and the quantity of chemerin isoforms were not determined.

Currently, there is little evidence for a physiologic or pathophysiologic role of huChem-163 in hepatocytes or hepatic stellate cells. The present study showed that hepatocyte released prochemerin, either by itself or after further processing, dampened inflammation of PBMCs, and, likewise, liver inflammation in an experimental NASH model. Thus, hepatocytes seem to provide prochemerin or so far unidentified chemerin isoforms to immune cells rather than reacting themselves to this multifunctional chemokine. This work provides evidence for a protective function of hepatocyte-produced prochemerin in experimental NASH.

## 5. Conclusions

Hepatocyte-produced chemerin lowered levels of inflammatory proteins in media of PBMCs and protected from hepatitis in a murine NASH model. Inflammation contributes to the development and progression of liver fibrosis, and strategies to elevate hepatocyte prochemerin levels may be protective.

## Figures and Tables

**Table 1:** Median, mean, and standard deviation of liver and spleen weight, serum adiponectin, glucose and insulin, and hepatic triglyceride and cholesterol levels (normalized to protein concentrations). None of these measures were changed by muChem-162 overexpression (*n* = 7 mice per group; Mann–Whitney U test). BW, body weight.

Measures	Median		Mean		Standard Deviation	
	C	162	C	162	C	162
Liver weight (g)	0.69	0.65	0.67	0.69	±0.10	±0.24
Spleen weight (g)	0.06	0.06	0.06	0.06	±0.001	±0.001
Adiponectin/BW (µg/mL/g)	0.48	0.35	0.53	0.33	±0.23	±0.18
Glucose/BW (mg/dL/g)	4.19	6.24	3.64	5.81	±2.14	±2.48
Insulin/BW (ng/mL/g)	0.02	0.01	0.02	0.02	±0.01	±0.007
Triglycerides/protein (nmol/g)	0.81	0.58	1.00	0.79	±0.61	±0.53
Cholesterol/protein (mg/g)	7.30	7.70	7.10	9.00	±5.00	±3.00

**Table 2:** Median, mean, standard deviation, and *p*-values of genes expressed in the liver of control (C) and muChem-162 (162)-overexpressing mice after feeding them an methionine–choline deficient (MCD) diet for 2 weeks. For normalization, 18S rRNA was used. Genes were ordered alphabetically. n.s., not significant. (*n* = 7 mice per group; Mann–Whitney U test).

Gene	Median		Mean		Standard Deviation		*p*-Value
	C	162	C	162	C	162	
*Alpha-SMA*	3.59	1.33	3.21	1.55	±1.22	±0.81	0.038
*CCL2*	1.17	0.68	1.30	0.61	±0.81	±0.21	n.s.
*CCL3*	1.12	0.26	1.29	0.34	±0.74	±0.19	0.009
*CCL7*	2.31	0.59	2.61	0.73	±0.82	±0.55	0.004
*CD38*	1.20	0.79	1.24	0.82	±0.29	±0.25	0.017
*CD68*	1.55	0.54	1.55	0.64	±0.59	±0.39	0.008
*CD163*	0.23	0.80	0.25	0.85	±0.08	±0.49	0.001
*Col1a1*	3.61	0.85	3.67	0.99	±2.16	±0.45	0.004
*CTGF*	1.29	0.68	1.19	0.75	±0.32	±0.23	0.035
*F4/80*	16.3	2.68	17.4	4.05	±8.86	±3.58	0.004
*IL-6*	1.19	0.81	1.33	1.04	±0.57	±0.64	n.s.
*Ly49C*	0.86	0.44	0.86	0.52	±0.39	±0.29	n.s.
*Ncr1*	1.29	0.43	1.22	0.54	±0.35	±0.28	0.010
*TGFβ*	2.61	1.07	2.19	1.18	±0.84	±0.42	0.026
*TNF*	0.99	0.42	1.14	0.47	±0.61	±0.21	0.017

## Data Availability

The data sets for this study can be obtained from the corresponding author.

## Supplementary Materials

The following are available online at https://www.mdpi.com/article/10.3390/biomedicines10010132/s1, Table S1: Antibodies used in the present study; Table S2: Primer used for real-time PCR; Table S3: Expression of different proteins in the liver of control and AAV8-muChem-162–transfected mice; Table S4: Activation of CMKLR1 and GPR1 by chemerin overexpressed in Hepa1–6 cells; Table S5: Ratios of phosphorylated to non-phosphorylated Stat3, Akt, p38, p65, and ERK in Hepa1–6 cells overexpressing muChem-162; Table S6: CCL2, TNF, and CXCL1 in cell media of Hepa1–6 cells at 48 h post-transfection; Table S7: CCL2, osteopontin, and IL-6 in cell media of LX-2 cells at 72 h post-transfection; Table S8: Osteopontin in cell media of Huh7 cells at 48 h post-transfection; Figure S1: Body weight before the start of the experiment and 1 and 2 weeks after feeding of the MCD diet; Figure S2: Cell media CCL20 levels of PBMCs cultivated in the supernatant of Huh7 cells transfected with a control plasmid or overexpressing huChem-163.
