# Supplementary material for: Chemerin Overexpression in the Liver Protects against Inflammation in Experimental Non-Alcoholic Steatohepatitis
Source: Biomedicines. 2022 Jan 7;10(1):132. doi: 10.3390/biomedicines10010132 (PMC8773259; doi:10.3390/biomedicines10010132)

Supplementary Table S1: Antibodies used in the present study.

Antigen	Company	Host	Order Number
4-Hydroxynonenal	R&D Systems	mouse	MAB3249-SP
Akt	Cell Signaling	rabbit	4691
Alpha-SMA	Abcam	mouse	ab7817
Caveolin-1	Cell Signaling	rabbit	3267
CCL2	R&D Systems	mouse	AF479
Chemerin (human/mouse)	R&D Systems	goat	AF2324/5
C-reactive protein	R&D Systems	goat	AF1829
Erk	Cell Signaling	mouse	9107
F4/80	New England Biolabs	rabbit	70076
Galectin-3	BD	mouse	556904
GAPDH	Cell Signaling	rabbit	2118
p38	Cell Signaling	rabbit	9212
p65	Cell Signaling	rabbit	8242
phospho-Akt (Ser473)	Cell Signaling	rabbit	4060
phospho-Erk (Thr202/Tyr204)	Cell Signaling	rabbit	4370
phospho-p38 MAPK (Thr180/Tyr182)	Cell Signaling	rabbit	4511
phospho-p65 (Ser536)	Cell Signaling	rabbit	3033
phospho-STAT3 (Tyr705)	Cell Signaling	rabbit	9145
SCD1	Cell Signaling	rabbit	2794
STAT3	Cell Signaling	mouse	9139

Supplementary Table S2: Primer used for real-time PCR.

Gene	Primer uni 5′→ 3′	Primer rev 5′→ 3′
18S rRNA	GATTGATAGCTCTTTCTCGATTCC	CATCTAAGGGCATCACAGACC
Alpha-SMA	CCAGCACCATGAAGATCAAG	CTTCGTCGTATTCCTGTTTGC
CCL2	AACTCTCACTGAAGCCAGCTCT	CTCTTGAGCTTGGTGACAAAAA
CCL3	TGCCCTTGCTGTTCTTCTCT	GTGGAATCTTCCGGCTGTAG
CCL5	TGCAGAGGACTCTGAGACAGC	GAGTGGTGTCCGAGCCATA
CCL7	TTCTGTGCCTGCTGCTCATA	TTGACATAGCAGCATGTGGAT
CD38	ACGCTGCCTCATCTACACTC	GGGGCGTAGTCTTCTCTTGT
CD68	TGATCTTGCTAGGACCGCTTA	CCCCTTGGACCTTGGACTA
CD163	GTTGTGACCGCCTGTATGAG	TTCCCAACTAGCTTTTCACCTC
Chemerin	AAACACCCACCTGTGCAGT	TTTTACCCTTGGGGTCCATT
Col1a1	CAGGGTCCTCCTGGTTCTC	GACCGTGAGTCCGTCTTTG
CTGF	CAAAGCAGCTGCAAATACCA	GGCCAAATGTGTCTTCCAGT
F4/80	TGCTCTTCCTGATGGTGAGA	CCCCGTCTCTGTATTCAACC
IL-6	CTCTGGGAAATCGTGGAAAT	CCAGTTTGGTAGCATCCATC
Ly49C	CCCTATTCCAGGGAGCTGT	TCTGTTTACCAGGAAGGAAGATG
Ncr1	TTGGCTCTTACAACGACTATGC	GTTGAAAGGTCAAACTCCCAAT
TGFβ	CTGGGCACCATCCATGAC	CAGTTCTTCTCTGTGGAGCTGA
TNF	CCGATGGGTTGTACCTTGTC	GGGCTGGGTAGAGAATGGAT

Supplementary Table S3: Expression of different proteins in the liver of control and AAV8-muChem-162 transfected mice. GAPDH was used as loading control. None of the measures were changed by muChem-162 overexpression. Mean values ± standard deviations are listed.

Protein	Control	muChem-162	Number of experiments
Caveolin-1	1.2 ± 0.6	1.1 ± 0.4	7
SCD1	1.3 ± 0.5	1.4 ± 0.3	7
p38	0.9 ± 0.4	1.0 ± 1.0	7
p-p38	1.4 ± 0.8	1.3± 1.1	7
p-p38/p38	1.9 ± 1.0	1.7 ± 0.7	7

Supplementary Table S4: Activation of CMKLR1 and GPR1 by chemerin overexpressed in Hepa1-6 cells. Median values are given in nM/ng. Not detectable, n.d.

Protein	Control	muChem-156	muChem-162	Number of experiments
huCMKLR1	n.d.	3.96	0.77	4
muCMKLR1	n.d.	2.56	1.36	4
huGPR1	n.d.	1.77	0.54	4

Supplementary Table S5: Ratios of phosphorylated to non-phosphorylated Stat3, Akt, p38, p65 and ERK in Hepa1-6 cells overexpressing muChem-162. Mean values ± standard deviations are shown. None of the measures were changed by muChem-162 overexpression

Protein	Control	muChem-162	Number of experiments
pStat3/Stat3	1.0 ± 1.4	0.4 ± 0.1	3
pAkt/Akt	0.2 ± 0.2	0.2 ± 0.2	3
p-p38/p38	0.4 ± 0.1	0.5 ± 0.1	5
p-p65/p65	0.3 ± 0.1	0.4± 0.3	3
p-ERK/ERK	0.7 ± 0.3	0.9 ± 0.6	3

Supplementary Table S6: CCL2, TNF and CXCL1 in cell media of Hepa1-6 cells at 48 h post-transfection. Mean values ± standard deviations are shown. None of the measures were changed by muChem-162 overexpression.

Cytokine / Chemokine	Control	muChem-162	Number of experiments
CCL2 (ng/ml)	3.6 ± 1.2	3.2 ± 0.1	3
TNF (pg/ml)	7.1 ± 5.0	7.5 ±3.7	3
CXCL1 (ng/ml)	2.9 ± 3.2	2.3 ± 2.1	4

Supplementary Table S7: CCL2, osteopontin and IL-6 in cell media of LX-2 cells at 72 h post-transfection. Mean values ± standard deviation are shown. None of the measures were changed by huChem-163 overexpression.

Cytokine / Chemokine	Control	huChem-163	Number of experiments

CCL2 (ng/ml)	1.6 ± 0.3	2.3 ± 1.1	3
IL-6 (pg/ml)	650 ± 320	1200 ±720	4
Osteopontin (pg/ml)	118 ± 26	132 ± 51	6

Supplementary Table S8: Osteopontin in cell media of Huh7 cells at 48 h post-transfection. Mean values ± standard deviations are shown. Osteopontin in cell media was not changed by huChem-163 overexpression.

Cytokine / Chemokine	Control	huChem-163	Number of experiments
Osteopontin (ng/ml)	1.9 ± 0.4	2.0 ± 0.6	4

Supplementary Figure S1: Body weight before the start of the experiment and 1 and 2 weeks after feeding the MCD diet. Mean values ± standard deviations are shown. Body weight between the two groups did not differ at any time point.

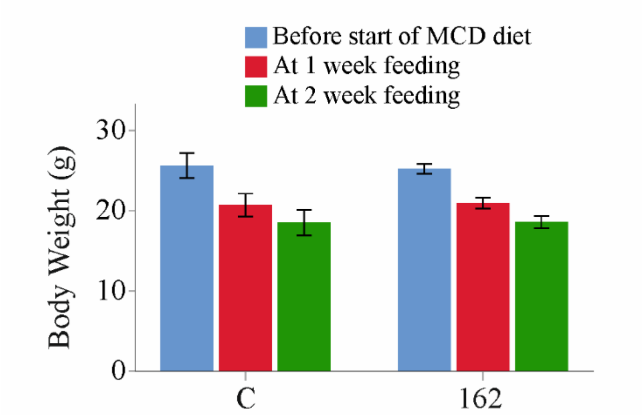

Supplementary Figure S2: Cell media CCL20 levels of PBMCs cultivated in the supernatant of Huh7 cells transfected with a control plasmid or overexpressing huChem-163, n = 4.

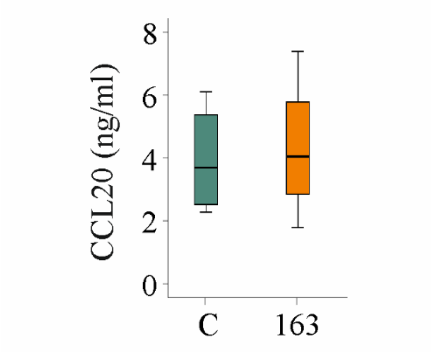

Supplement: Supplementary file 1 [file biomedicines-10-00132-s001.zip › biomedicines-1494175-supplementary.pdf]
